# Visual-spatial information processing in the two hemispheres of the brain is dependent on the feature characteristics of the stimulus

**DOI:** 10.3389/fnins.2013.00010

**Published:** 2013-02-01

**Authors:** Kenneth Hugdahl

**Affiliations:** ^1^Department of Biological and Medical Psychology, University of BergenBergen, Norway; ^2^Department of Radiology, Haukeland University HospitalBergen, Norway; ^3^Division of Psychiatry, Haukeland University HospitalBergen, Norway

**A commentary on**

**Global versus local processing: seeing the left side of the forest and the right side of the trees**

by Christie, J., Ginsberg, J. P., Steedman, J., Fredriksson, J., Bonilha, L., and Rorden, C. (2012). Front. Hum. Neurosci. 6:28. doi: 10.3389/fnhum.2012.00028

## Hemispheric asymmetry for visual processing

The two hemispheres of the brain differ in their capacity for processing of information, with the left hemisphere being specialized, or dominant, for language processing and the right hemisphere being specialized or dominant for processing of visual-spatial relations (see Davidson and Hugdahl, 1993; Hugdahl, 2010 for overviews of research on hemispheric asymmetry, or laterality).

**Figure 1:**
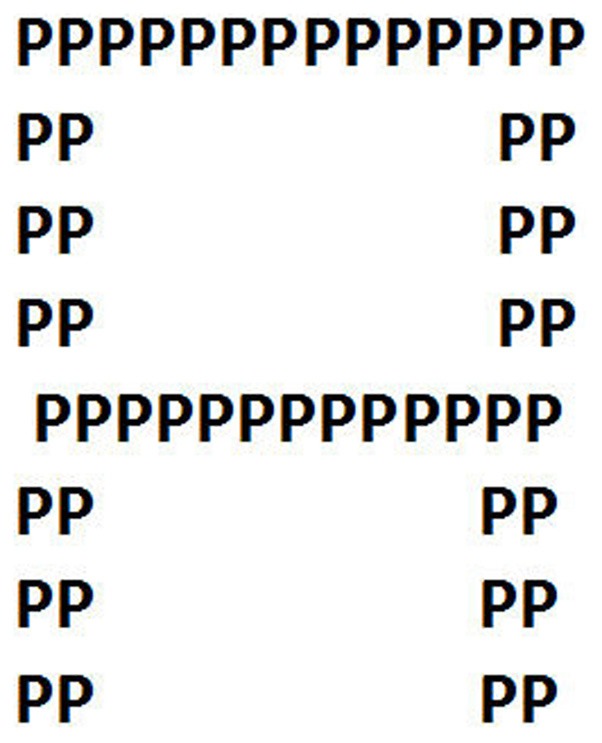
**Illustration of local and global processing elements in a visual stimulus.** The global element (letter A) is made up of local elements (letter P). The stimuli are flashed briefly to the left or right hemisphere, and the subject is instructed to either identify the global or local element on a trial.

A particular aspect of hemispheric asymmetry is asymmetry for processing of objects presented in the left or right visual half-field (see Hellige et al., 2010 for a recent overview of asymmetry for visual processing). This technique involves visual stimuli that are briefly flashed either to the right or left side of a centrally placed fixation point in the visual field. Provided that the subject or patient is fixating in the middle of the visual field, stimuli in the right half-field will be initially projected to the left visual cortex, and vice versa for stimuli in the left half-field. This is so because the optical projections from the retina to the visual cortex are arranged such that light falling onto the nasal region of the retina of both eyes will project contralaterally, while light falling onto the lateral region of the retina will project ipsilaterally. This will result in a contralateral projection of light in the two visual half-fields, given that the subject is fixating a point in the middle of the visual field. Using an ingenious paradigm with single letters that were made up of smaller letters, that could be the same or different as the large letters, Navon (1977) used half-field stimulus input, and found that while the left hemisphere was more sensitive to the local elements of the stimulus (small letters), the right hemisphere was more sensitive for global element (large letter). Figure 1 shows an example of a large letter (A) being made up of smaller letters (P).

Based on the original findings by Navon, others, e.g., Robertson and Ivry (2000) and Sergent (1982) formulated theoretical models of how the two hemispheres of the brain processed local and global elements of a visual stimulus.

## A paradox resolved—egocentric vs. allocentric processing

However, as pointed out in the current study by Christie et al. (2012) despite clear hypotheses of how the hemispheres should process global and local stimulus elements, nevertheless subtle differences between the hemispheres for local and global processing have been found over the years. An explanation for this could, according to the authors, be found in studies of patients with spatial neglect (a condition after stroke that affects the patient's ability to attend to and identify objects in the left or right visual half-field). Studies of these patients have shown that while right hemisphere lesions result in a condition called egocentric neglect, i.e., losing the ability to identify objects presented in the left visual half-field, left hemisphere lesions result in a condition called allocentric neglect, i.e., losing the ability to identify the right side of objects irrespective of the side in space they are presented. The authors then go on and suggest that this may also explain why effects are subtle in experimental studies on healthy subjects, because researchers have not distinguished between these fundamentally different forms of lateral bias for visual processing. The authors suggest the interesting hypothesis that the hemispheres differ in their sensitivity for allocentric processing irrespective of differences for egocentric processing. They suggest that while the left hemisphere process the right side of objects, the right hemisphere process the left side of objects independent of the hemi-space the objects is seen in. They tested their hypothesis in both a group of healthy individuals and in a patient with a left-sided lesion (stroke). The authors designed a novel experimental paradigm which consisted of geometric shapes (circles, squares, and triangles) made up of smaller shapes that were presented either on a global, or allocentric and egocentric local scales.

The task of the subject was to press a button whenever a circle (target stimulus) was present in a display irrespective of if it occurred on the local or global scale level.

## Confirming the hypothesis

The results showed a significant interaction between side of target stimulus presentation and scale, such that targets were detected with higher accuracy for left side presentations at the global scale of processing and higher accuracy for right side presentations at the local scale of processing. The results for the neurologically intact subjects were mimicked in the neurologically damaged patient, although he showed a larger difference between left and right target localization for local scale processing, which made the authors comment that perhaps the underlying mechanisms are not identical between the subject categories.

## New knowledge for an old issue

Although the present results basically shed new light on an old issue in laterality research, i.e., how global and local items of a complex visual stimulus are processed by the two hemispheres of the brain, the findings may also have implication for our understanding of visual processing in patients after unilateral brain lesions. Such patients have problems with identifying objects in the half-field contralateral to the lesioned hemisphere. This is more common after right hemisphere lesions, and such patients also have problems perceiving the global form of objects. Interestingly, patients with left hemisphere lesions often have problems perceiving and identifying the local details of objects.

## Better tests for neglect patients

The present results show that the difference in how right- vs. left-hemisphere lesions affect global and local processing falls back on a fundamental difference in processing capacity of the two hemispheres for global and local stimulus features. As such the results provide new insight into understanding the cognitive sequels of brain lesions, typically stroke, affecting primarily either the right or left hemisphere. The results also point to the possibility to test patients with the paradigm used in the present study to obtain a more fine-grained and detailed analysis of deficits in visual processing after unilateral lesions.
